# Protocol for a systematic review of the impact of resuscitation fluids on the microcirculation after haemorrhagic shock in animal models

**DOI:** 10.1186/s13643-015-0113-4

**Published:** 2015-10-05

**Authors:** David N. Naumann, Janine Dretzke, Sam Hutchings, Mark J. Midwinter

**Affiliations:** National Institute of Health Research, Surgical Reconstruction and Microbiology Research Centre, Queen Elizabeth Hospital, Birmingham, B15 2TH UK; School of Health & Population Sciences, College of Medical & Dental Sciences, University of Birmingham, Birmingham, UK; Kings College Hospital NHS Foundation Trust, London, UK

**Keywords:** Microcirculation, Haemorrhagic shock, Resuscitation

## Abstract

**Background:**

Modern resuscitation strategies following haemorrhagic shock are influenced by global haemodynamic parameters such as blood pressure and cardiac output. Microcirculatory dysfunction in this context may persist even after restoration of satisfactory global parameters. Additional monitoring of the microcirculatory function may therefore be warranted in order to facilitate goal-directed therapy at a tissue oxygenation level. Although such a phenomenon is recognised in the case of sepsis, clinical evidence regarding the behaviour of the microcirculation following the delivery of resuscitation fluids after haemorrhagic shock is sparse. A summation of the current state of pre-clinical evidence is justified in order to direct avenues for future clinical research.

**Methods/design:**

Systematic review methodology will be utilised in order to identify relevant studies, assess for bias, and extract data for analysis. Medical databases will be searched to find pre-clinical studies that monitor the microcirculatory function following haemorrhagic shock and subsequent fluid resuscitation. Different fluid types (e.g. blood products, crystalloid, and colloid fluids) will be compared. The search strategy will combine terms for the animal model, resuscitation fluid, and microcirculatory parameters. Randomised and non-randomised experiments, as well as case series, will be eligible for inclusion. Specific quality assessment tools for pre-clinical research will be used depending on study design. A combination of narrative and meta-analysis techniques will be used for the synthesis of data.

**Discussion:**

The choice of type, sequence, and quantity of resuscitation fluid following haemorrhagic shock is controversial, and the optimal strategy for restoration of microcirculatory function is yet unknown. A detailed examination of pre-clinical data regarding the microcirculation is timely and will enable a focussed approach to clinical research for the improvement of resuscitation following haemorrhagic shock.

**Systematic review registration:**

Collaborative Approach to Meta Analysis and Review of Animal Data from Experimental Studies (CAMARADES)

**Electronic supplementary material:**

The online version of this article (doi:10.1186/s13643-015-0113-4) contains supplementary material, which is available to authorized users.

## Background

Global measures of circulatory function such as cardiac output have long been used in the targeted resuscitation of trauma casualties, with the focus on maintaining parameters of measurements such as blood pressure and heart rate. Such parameters are really only surrogate markers of tissue perfusion since they do not directly describe the transfer of oxygen and nutrients between the circulation and tissues but instead concern more global transfer of blood around the body. Since shock can be described as *inadequate oxygenation of tissues due to circulatory failure*, it may be more appropriate to examine the end vessels that directly participate in this process—the microcirculation. The microcirculation can be described as the network of small vessels such as capillaries that perform the physiological functions of oxygen delivery and respiratory substrates to the cells. Therefore, the microcirculation may represent the effectiveness of oxygen delivery better than the macro-circulation.

There is a clinical correlation between microcirculatory dysfunction and poorer patient outcomes in sepsis [1], and early pilot data has suggested the same for patients with traumatic haemorrhagic shock [2]. Outcomes appear to be improved when goal directed treatment is aimed at the restoration of the microcirculation in the case of sepsis [3] and after major surgery [4]. Some patients may have critically impaired microcirculation even when the more global measurements such as cardiac output appear to be well controlled, and therefore, targeted resuscitation might be improved with emphasis on the microcirculation rather than more conventional and commonly measured global markers of haemodynamics. If such a phenomenon proves true for haemorrhagic shock, then a greater understanding of microcirculatory changes following delivery of resuscitation fluids may open channels for future therapeutic trials into goal directed therapy for trauma patients.

So far, there has been no systematic review of pre-clinical data with regard to resuscitation fluids and the microcirculation. The Microshock study (ClinicalTrials.gov identifier: NCT02111109) aims to characterise the microcirculatory changes in patients with traumatic haemorrhagic shock, but the specific effects of resuscitative fluids on the microcirculation is unknown. It is timely to conduct a systematic review to summarise the available data in the published pre-clinical literature regarding the impact of resuscitation fluids on the microcirculatory function following haemorrhagic shock. This will provide exploratory data to generate new hypotheses that might direct further avenues for clinical research.

## Methods

This protocol was written using the SYRCLE format [5]. Standardised systematic review methodology will be utilised according to the Cochrane handbook [6] and reported according to the Preferred Reporting Items for Systematic Reviews and Meta-Analyses (PRISMA) guidelines [7]. Where relevant, reporting guidelines developed for systematic reviews of pre-clinical studies will also be used [8].

### Research questions

This is the first review to examine the impact of different fluid resuscitation techniques on the physical structure and function of the microcirculation in animal models. The following questions will be addressed:

- What is the impact of intravascular fluid resuscitation on the microcirculation following haemorrhagic shock, compared to haemorrhagic shock alone?
- Which type of fluid has the most impact on the microcirculation following haemorrhagic shock, compared to haemorrhagic shock alone?
- Compared to crystalloid fluid, which type of fluid has more of an impact on the microcirculation: colloid or packed red blood cells (PRBCs)?

### Disease of interest

The pathophysiological process of interest is haemorrhagic shock.

### Population studied

This systematic review will examine all animal models of haemorrhagic shock, regardless of mode of haemorrhage, species, or size of animal.

### Intervention

Exploratory assessment of relevant studies has revealed that there are multiple interventions of interest. For example studies may test (i) blood products versus crystalloids versus haemorrhage alone or (ii) crystalloids versus colloids versus haemorrhage alone in the resuscitation of animals following haemorrhagic shock.

In order to address this heterogeneity, and still provide an informative summary of all intravascular fluid resuscitation techniques, each individual intervention will analysed as subgroups to test the individual as well as summary effects. This will be in the form of meta-analytical techniques when there are sufficient numbers of studies with homogenous design and, in more qualitative terms, when studies are heterogeneous and small in number. The following interventions are determined a priori:

- Primary intervention: PRBCs
- Secondary interventions: whole blood; plasma; colloids (e.g. 10 % hydroxyethyl starch, albumin); crystalloids (e.g., Ringer’s lactate, normal saline); modified blood components (e.g. PRBC + nitrites or RRx-001)

PRBC has been chosen as the primary intervention because it is the most likely intravascular resuscitative measure delivered in a clinical setting for patients who have suffered traumatic haemorrhagic shock.

### Control population

The control population for meta-analysis will be animals that have undergone the haemorrhagic shock protocol alone, with no intervention. Studies comparing different types of fluids but without haemorrhagic shock alone will be included but analysed separately. Non-controlled studies will also be eligible for inclusion but for descriptive analysis only.

### Outcome measures

Preliminary searches have shown that there are multiple microcirculatory physical parameters that have been reported in experimental studies of fluid resuscitation following haemorrhagic shock. The following outcome measures (measured at any anatomical position, provided that it is in the microcirculatory vessels) will be used:

- Primary outcome: flow rate (nL/s)
- Secondary outcomes: vessel diameter (μm); functional capillary density (%); red blood cell velocity (mm/s); shear rate (s^−1^); glycocalyx thickness (μm); permeability (%); proportion of perfused vessels (%)

Since this review is primarily concerned with the pathophysiology of the microcirculation, clinical and prognostic factors of the animal models will not be examined in this review. This is because the animals are likely to be euthanized before longer-term data is available.

### Search and study identification

The following sources will be searched for primary studies: EMBASE and Medline (via OVID SP) and SCOPUS. Before starting this review, scoping searches were undertaken to identify any existing systematic reviews of the impact of resuscitative fluids on the microcirculatory changes during haemorrhagic shock and to gauge the number and type of relevant primary studies. No systematic reviews were identified, but there are a number of pre-clinical studies.

The systematic search will be undertaken using a combination of text and MeSH terms relating to the intervention (“bleed”, “transfusion”, “fluid resuscitation”, “colloid”, “red blood cells”), to the condition (e.g. “haemorrhage”, “trauma”, “injury”, “shock”), and to the microcirculation (e.g. “microcirculation”, “endothelium”, “capillary”); see Additional file 1 for a sample search strategy in MEDLINE. The search strategies will be broad in order to capture any study that has explored resuscitation fluids and microcirculatory changes in the context of haemorrhagic shock, regardless of study design, study subjects, method of assessment, or outcomes reported. There will be no language restrictions or restrictions by publication type. Citations will be collated using EndNote reference management software (V.X7, Thomson Reuters). Reference lists of included studies and relevant reviews will be screened for further eligible studies.

### Study selection

#### Screening phases

All titles and abstracts will be screened for eligibility by two independent reviewers, and full texts obtained for potentially relevant studies. References from these full texts will be further screened for potentially relevant papers using pre-defined selection criteria. The study selection process will be illustrated using a PRISMA flow diagram.

#### Reviewers and discrepancies

All titles and abstracts will be screened for eligibility by two authors. Discrepancy between reviewers will be resolved by discussion or by referring to a third reviewer.

### Inclusion and exclusion criteria

#### Type of study (design)

Any pre-clinical study may be eligible for inclusion if it reports data regarding the impact of resuscitation fluids on the microcirculation in an animal model that has been subject to any form of haemorrhagic shock. These may include randomised controlled trials (e.g. where microcirculatory dynamics are an outcome measure to assess the effect of alternative resuscitative fluid interventions for haemorrhagic shock) or prospective case series or controlled studies (e.g. where haemorrhagic shock is induced in animals and effects on the microcirculation monitored following fluid resuscitation). Single case reports, conference proceedings, abstracts, and letters to the editor will be screened but will be subsequently excluded if essential methodological information is missing.

#### Type of animal/population

All animal models of haemorrhagic shock are eligible for inclusion, regardless of gender, age, or species. Knockout animals and those with co-morbidities are eligible for inclusion but will be analysed separately (i.e. not pooled with wild-type or healthy animals).

#### Type of intervention

Animals must receive intravascular fluid of any volume in order to be eligible for inclusion, which may include any of whole blood, packed red blood cells, plasma, crystalloid fluid, colloid fluid, altered blood products, or a combination of these. The fluid must be delivered after a phase of haemorrhagic shock and subsequent microcirculatory measurements taken following its delivery. Studies that report data regarding the microcirculation will be excluded if this is not in the context of haemorrhagic shock (e.g. studies that only discuss sepsis will be excluded). Studies reporting both cases of sepsis and haemorrhagic shock will be included providing data can be separated for haemorrhagic shock only.

#### Outcome measures

Studies are included if they record at least one physical parameter of the microcirculation following a phase of haemorrhagic shock. Studies that only report microcirculatory function (such as oxygen pressures) will be excluded unless they also report the physical properties.

#### Restrictions

There are no language restrictions. Where necessary, translation (full/part) of non-English language articles will be undertaken. No publication date restriction will be applied. Although the pathophysiological process of interest is traumatic haemorrhagic shock, any model of haemorrhagic shock will be eligible for inclusion. This includes models of obstetric haemorrhage in pregnant animals. Where studies are found that also model trauma (such as blast or penetrating injury), these will be highlighted and analysed separately.

#### Exclusion criteria by stage

Exclusion of type of intervention and outcome measures will be conducted during title/abstract screening. Any further exclusion will be made following the full text screening.

### Study characteristics to be extracted

Data from all relevant full texts will be extracted using a standardised, piloted data extraction form. Data extraction will be performed by one reviewer and checked by a second. Study authors will be contacted where information in the published articles is missing or unclear.

#### Study ID

The first author and year will be extracted to identify individual studies. Where multiple studies have the same author and year, the month of publication will also be extracted.

#### Study design characteristics

Design and methodology of individual studies will be extracted, including type of study, sample size, experimental groups, animal housing, and length of follow-up.

#### Animal model characteristics

Species of animal as well as age, strain, weight, sex, and genetic modification factors will be extracted. The exact protocol for haemorrhagic shock will be extracted, including volume of blood, percentage of blood, as well as the timings of shock and intervention. Techniques, equipment, and measurements of measuring the microcirculatory parameters will be extracted, including anatomical region.

#### Intervention characteristics

Type of fluid (e.g. red blood cells, whole blood, altered blood, plasma, platelets, crystalloid, and colloid fluids) will be extracted, as well as volume, rate, and timings.

#### Outcome measures

All findings relating to microcirculatory parameters will be extracted, as well as physiological parameters such as mean arterial pressure, blood pressure, and heart rate.

#### Other parameters

Number of deaths, dropouts, and incomplete experiments will also be extracted.

### Assessment of risk of bias and study quality

#### Reviewers and discrepancies

Risk of bias will be assessed by two authors. Discrepancy between authors will be resolved by discussion or by referring to a third until consensus achieved.

#### Study assessment

Animal intervention studies will assessed using the Systematic Review Centre for Laboratory animal Experimentation (SYRCLE) risk of bias tool, which includes domains for selection bias (sequence generation, baseline characteristics, allocation concealment), performance bias (random housing and blinding), detection bias (random outcome assessment and blinding), attrition bias, reporting bias, and other biases [9]. Case series will be assessed based on the criteria proposed by Moga et al. and tailored as appropriate to the subject area [10].

Attention will also be paid towards the validity of design and conduct of the studies and the translatability to humans, according to the summary domains highlighted by Henderson et al. following their examination of 26 separate guidelines [11]. This will be discussed in depth following analysis of the data extracted.

### Collection of outcome data

#### Type of outcome data to be extracted

Outcome data, units, and type (continuous/dichotomous) will be as follows:

- Blood flow (nL/s); continuous data
- Red cell velocity (mm/s); continuous data
- Wall shear rate (s^−1^); continuous data
- Glycocalyx and vessel thickness (μm); continuous data
- Microvascular permeability (%); continuous data
- Functional capillary density (%); continuous data
- Perfused microvessel density (n/mm); continuous data
- Proportion of perfused vessels (%); continuous data

#### Method of data extraction

Primary method for data extraction will be taking the numerical values directly from the results sections and tables of individual studies. The three values of interest for each outcome are the mean, standard deviation (SD), and number of animals (*N*).

Where only percentages are reported in the place of raw numerical data, the numerical values will be calculated based on the reported percentage and denominator. Where a numerical value is missing but the data is displayed graphically, a digital screen ruler will be used to extract the numerical data. Authors will be contacted where these techniques are not possible.

#### Reviewers and discrepancies

Outcome data extraction will be performed by one reviewer and checked by a second.

### Data analysis and synthesis

#### Combining and comparing the data

A combination of meta-analysis and descriptive techniques will be used to report the outcome data. The technique used will depend on the homogeneity and number of studies that can be feasibly pooled together for synthesis. Meta-analysis will be conducted using Stata data analysis software (V14, StataCorp LP). Where a number of similar studies (in terms of animal model, intervention and outcomes) are reported by the same study group or authors over time, authors will be contacted to determine whether studies are independent or include previously reported data.

#### Feasibility of meta-analysis

An assessment of clinical and methodological heterogeneity will be undertaken in order to inform a decision on whether to pool data. Studies will be considered homogeneous if they have a similar model of haemorrhagic shock, use similar methods in measuring the microcirculatory parameters, test the same interventions, and measure the same outcomes.

Where studies are considered too heterogeneous to pool, synthesis will be narrative, and forest plots may be used for illustration of heterogeneity without pooling.

#### Effect measures

All of the outcomes of interest are continuous variables but may have been measured using different instruments and in different sizes of animals. Where it is not possible to pool results using the mean difference, the normalised mean difference (NMD) and standard deviation will be used in meta-analysis as described by Vesterinen et al. [12].

The actual effect size in animal studies does not necessarily translate to humans. Therefore, rather than concentrate on the actual size of effect, emphasis will be placed on the direction and consistency of effect, and exploration of any heterogeneity.

#### Statistical modelling

Preliminary searches have demonstrated a relatively high level of heterogeneity between studies. Underlying effect size is likely to differ between studies even where there is a degree of methodological homogeneity. Therefore, where meta-analysis is used, random effects modelling will be utilised in order to measure the mean of a distribution of effects and to account for residual heterogeneity.

#### Statistical methods to assess heterogeneity

The presence of heterogeneity will be assessed by visual inspection of forest plots, as well as with the chi-squared statistic. The *I*^2^ statistic will be used to quantify the percentage of variability due to heterogeneity. Heterogeneity will be categorised as suggested in the Cochrane guidelines (http://handbook.cochrane.org) as unimportant (0–40 %); moderate (30–60 %); substantial (50–90 %), and considerable (75–100 %).

#### Subgroup analysis

Subgroup analysis will be performed for each intervention (i.e. whole blood, PRBC, plasma, colloid, crystalloid, altered blood products). Further subgroup analysis of different species of animal and different outcome measures will be conducted. The findings from subgroup analyses will be interpreted cautiously and used for generating hypotheses only, rather than drawing more general conclusions. Appropriate caveats will be made when reporting these results, and *p* values will not be used.

#### Sensitivity analysis

Issues that require sensitivity analysis include the age of study, as well as only including studies that were at low risk of bias according to the SYRCLE risk of bias tool. Other sensitivity analyses may relate to which analysis methods were used (e.g. change in values versus final values for continuous data or based on how missing data had been handled).

#### Other meta-analysis details

In addition to pairwise meta-analysis of individual interventions versus control, further multi-treatment meta-analysis will be performed for simultaneous comparison of multiple interventions, as described by Caldwell et al. [13]. The multiple interventions to be compared will include PRBC versus crystalloid versus colloid.

#### Publication bias

Publication bias may be a particular risk with pre-clinical/animal studies [14, 15]. For each meta-analysis containing 20 or more studies, the likelihood of publication bias will be investigated through the construction of funnel plots and appropriate statistical tests for small-study effects (such as the Peter’s Test [16]), that is, the tendency for smaller studies to provide more positive findings. It is well-recognised that, especially where heterogeneity exists, publication bias may be one of a number of reasons for any small-study effects identified.

## Discussion

The optimal volume, ratios, type, sequence of delivery, and timing of resuscitation fluids following haemorrhagic shock of trauma remain controversial. Low volume resuscitation has been advocated following trauma in order to reduce haemodilation and the effects of trauma-induced coagulopathy [17]. However, strategies which aim for a certain mean arterial pressure or which utilise ‘permissive hypotension’ may not take into account the significance of the physical dynamics of the microcirculatory oxygen exchange surface. Since the primary aims of resuscitation following haemorrhagic shock are to restore tissue oxygenation, repay oxygen debt, and reduce acidosis, it is the microcirculation that is the key ‘target organ’ in this process. It is possible that the monitoring of the function of the microcirculation may have a role in the future as a means by which resuscitation can be optimised [18].

In order to plan and conduct clinical investigation of the microcirculation following haemorrhagic shock and fluid resuscitation, it is first advisable to examine all the available pre-clinical evidence. This may enable greater focus and attention towards certain avenues and research questions of interest. This systematic review will examine the pre-clinical evidence available with regard to microcirculatory function following resuscitation in the context of haemorrhagic shock and will propose clinical questions of interest based on the findings.

## Additional file

Additional file 1:
**Sample search strategy in MEDLINE.** This search strategy was used to obtain the details for potentially relevent studies of interest.

## Abbreviations

CAMARADES: Collaborative Approach to Meta Analysis and Review of Animal Data from Experimental Studies
CI: confidence interval
PRBC: packed red blood cells
PRISMA: Preferred Reporting Items for Systematic Reviews and Meta-Analyses
SD: standard deviation
SMD: standardised mean difference
SYRCLE: Systematic Review Centre for Laboratory animal Experimentation
