# Supplementary material for: Protocol for a systematic review of the impact of resuscitation fluids on the microcirculation after haemorrhagic shock in animal models
Source: Syst Rev. 2015 Oct 5;4:135. doi: 10.1186/s13643-015-0113-4 (PMC4593218; doi:10.1186/s13643-015-0113-4)
Supplement: Additional file 1: — Sample search strategy in MEDLINE. This search strategy was used to obtain the details for potentially relevent studies of interest. [file 13643_2015_113_MOESM1_ESM.doc]

**Additional File 1. Sample search strategy in MEDLINE**

Database: Ovid MEDLINE(R) In-Process & Other Non-Indexed Citations and Ovid MEDLINE(R) <1946 to Present>

Search Strategy:

--------------------------------------------------------------------------------

1 haemorrhag$.mp. (42370)

2 hemorrhage.mp. or exp Hemorrhage/ (317823)

3 bleed$.mp. (152764)

4 trauma.mp. or exp "Wounds and Injuries"/ (815484)

5 shock.mp. or exp Shock, Hemorrhagic/ or exp Shock, Traumatic/ or exp Shock/ (188261)

6 microcirculation.mp. or exp Microcirculation/ (41855)

7 microcirculat$.mp. [mp=title, abstract, original title, name of substance word, subject heading word, keyword heading word, protocol supplementary concept word, rare disease supplementary concept word, unique identifier] (44370)

8 capillar$.mp. or exp Capillaries/ (144927)

9 exp Endothelium/ or endothelium.mp. (149398)

10 1 or 2 or 3 or 4 or 5 (1342437)

11 7 or 8 or 9 (308688)

12 10 and 11 (21552)

13 (fluid$ adj3 resuscitat$).mp. [mp=title, abstract, original title, name of substance word, subject heading word, keyword heading word, protocol supplementary concept word, rare disease supplementary concept word, unique identifier] (4445)

14 (fluid$ adj3 administrat$).mp. [mp=title, abstract, original title, name of substance word, subject heading word, keyword heading word, protocol supplementary concept word, rare disease supplementary concept word, unique identifier] (3158)

15 normal saline.mp. (15704)

16 exp Fluid Therapy/ or crystalloid$.mp. (20028)

17 (colloid$ adj3 resuscitat$).mp. [mp=title, abstract, original title, name of substance word, subject heading word, keyword heading word, protocol supplementary concept word, rare disease supplementary concept word, unique identifier] (212)

18 (red blood cell$ or RBC or plasma or platelet$ or whole blood or blood product$ or blood component$).mp. [mp=title, abstract, original title, name of substance word, subject heading word, keyword heading word, protocol supplementary concept word, rare disease supplementary concept word, unique identifier] (1033868)

19 exp Blood/ (962483)

20 18 or 19 (1806182)

21 (resuscitat$ or transfus$).mp. [mp=title, abstract, original title, name of substance word, subject heading word, keyword heading word, protocol supplementary concept word, rare disease supplementary concept word, unique identifier] (182117)

22 20 and 21 (54184)

23 13 or 14 or 15 or 16 or 17 or 22 (91548)

24 12 and 23 (1126)
